# *ITPKC* polymorphism (rs7251246 T > C), coronary artery aneurysms, and thrombosis in patients with Kawasaki disease in a Southern Han Chinese population

**DOI:** 10.3389/fimmu.2023.1184162

**Published:** 2023-06-19

**Authors:** Jie Liu, Piaoliu Yuan, Yusheng Pang, Danyan Su

**Affiliations:** Department of Pediatrics, First Affiliated Hospital, Guangxi Medical University, Nanning, China

**Keywords:** Kawasaki disease, coronary artery aneurysm, thrombosis, ITPKC, polymorphisms

## Abstract

**Objectives:**

Kawasaki disease (KD) is a commonly acquired pediatric systemic vasculitis disease resulting in coronary artery aneurysm (CAA). The relationship between the *ITPKC* polymorphism (rs7251246) and the severity and susceptibility to KD in the Han Chinese population in Southern China remains unclear.

**Methods:**

We enrolled 262 children as controls and 221 children with KD (46 [20.8%] with intravenous immunoglobulin resistance and 82 [37.1%] with CAA). The relationship between the *ITPKC* rs7251246 polymorphism, KD susceptibility, and CAA formation was investigated.

**Results:**

While the *ITPKC* rs7251246 T>C polymorphism was not significantly associated with KD susceptibility, it was significantly related to the CAA risk in children with KD [CC/CT vs. TT: adjusted odds ratio [OR] 2.089, 95% confidence interval [CI] 1.085–4.020]. Male children with the rs7251246 CT/TT genotype had a significantly lower risk of thrombosis [CT/TT vs. CC: adjusted OR 0.251, 95% CI 0.068–0.923]. Children with KD, especially those with CAA, had significantly downregulated *ITPKC* mRNA compared to healthy children. *ITPKC* mRNA levels were lower in children with CAA who developed thrombosis (*P*=0.039). In children with KD, the CC genotype showed lower mRNA levels of *ITPKC* (*P*=0.035).

**Conclusion:**

The *ITPKC* rs7251246 T>C polymorphism may be a risk factor for CAA and thrombosis in children with KD in the Han Chinese population, likely due to differences in mature mRNA levels caused by interference of RNA splicing. Dual antiplatelet therapy for thrombosis is recommended for male children with the rs7251246 CC genotype.

## 1 Introduction

Kawasaki disease (KD) is an acute self-limiting systemic vasculitis initially reported by Tomisaku Kawasaki in 1967 (1). KD has gradually become the most common cause of acquired heart disease in childhood (2–4). Although the etiology of KD remains unknown, it has been reported to be associated with the combined effects of infection, immune dysregulation, and genetic susceptibility. Epidemiological studies have reported that the incidence rates of KD vary among different regions, being much higher in Asian countries than in Western countries and increasing in Asian countries (especially in China) (5–7). Furthermore, male predominance and familial clustering suggest that hereditary factors may play an important role in KD occurrence and development (3, 8, 9). A genetic variation in the intracellular signaling pathway involved in immune effector function may increase the risk of KD and coronary artery disease (10, 11). Several networks may play a role in KD pathogenesis, and several susceptibility genes have been identified through genome-wide association and linkage studies. They can be classified into four groups according to their function: enhanced T cell activation, dysregulated B cell signaling, altered transforming growth factor beta signaling, and decreased apoptosis (12). In addition, a growing number of studies have demonstrated that some single-nucleotide polymorphism (SNP) gene loci have a close relationship with susceptibility to KD, the severity of KD, response to intravenous immunoglobulin (IVIG) treatment, and development of coronary artery complications (13–16).

The genetic association between KD and inositol 1,4,5-trisphosphate 3-kinase C (*ITPKC*) in a genome-wide scan was first reported by Onouchi et al. in 2008 (17). *ITPKC*, one of three isoenzymes of the ITPK family, located on chromosome 19q13.2, is responsible for the phosphorylation of inositol phosphate 3 (IP3) to 1,3,4,5-tetrakisphosphate (17); moreover, it is also involved in the Ca^2+^/nuclear factor of activated T cells (Ca^2+^/NFAT) pathway that negatively regulates T cell activation (17). A mutation in *ITPKC* can cause defective regulation of T cell activation (18, 19). Studies have shown that *ITPKC* is a crucial contributor to KD susceptibility and is associated with coronary artery aneurysm (CAA) formation (17, 20–23). Hence, *ITPKC* has been proposed as a susceptibility gene for KD, and its polymorphisms have become a research focus, as they may govern mRNA and protein *ITPKC* levels. This is consistent with *ITPKC* mutation and KD risk being negatively correlated (17). A functional SNP (rs28493229) located in intron 1 of *ITPKC* interferes with RNA splicing to affect transcriptional levels of mature mRNA, which is associated with KD susceptibility and the development of coronary artery lesions (CALs) in Japanese and American populations (17, 24). However, contrasting results were obtained in initial replication studies in the Taiwanese population (25, 26) and the Han population in mainland China (27). Furthermore, another SNP (rs2290692) was reported to be associated with susceptibility to KD in a Han Chinese population by Peng et al. in 2012 (21), which was also shown in a Taiwanese population with KD and CALs formation two years later (28). This study also suggested that a novel SNP (rs7251246) located in intron 1 of *ITPKC* may be used as a potential marker of KD severity in Taiwanese patients (28). Nevertheless, the mechanisms involved in the effect of rs7251246 on *ITPKC* expression remain unclear. Additionally, current research on the impact of the rs7251246 polymorphism on KD in the Han Chinese population in Southern China is lacking. Therefore, in the present study, we aimed to explore the association between the *ITPKC* (rs7251246) gene polymorphism and the susceptibility to KD in a Han Chinese population. We also explored the relationship between IVIG treatment response, CAA formation, immune/inflammation-related indicators, and genetic *ITPKC* polymorphisms. Furthermore, the *ITPKC* mRNA expression levels in the peripheral blood and potential mechanisms of action of the rs7251246 polymorphism were also investigated.

## 2 Materials and methods

### 2.1 Study population

**Figure 1:**
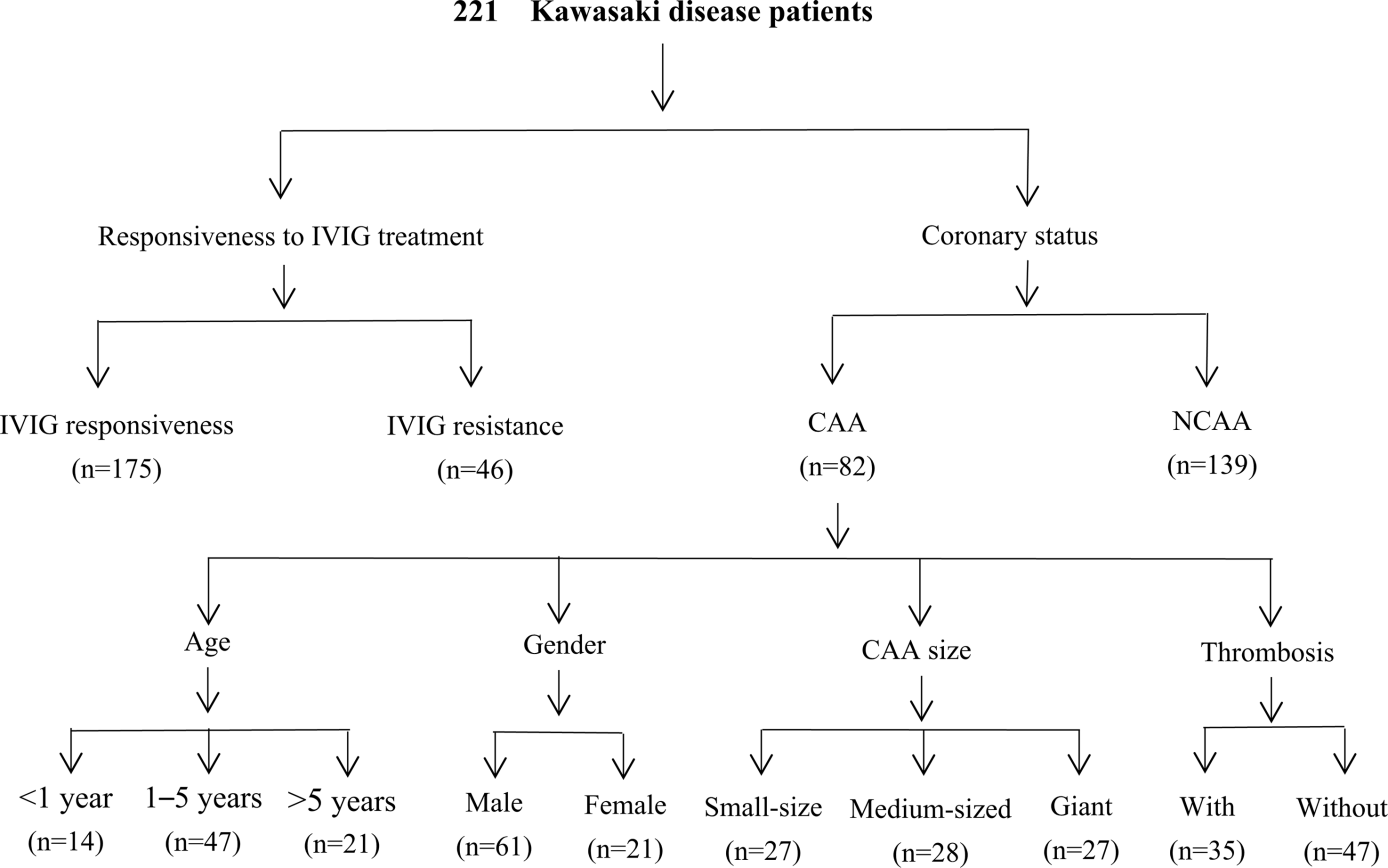
Flowchart of the study. IVIG, intravenous immunoglobulin; CAA, coronary artery aneurysm; NCAA, no coronary artery aneurysm.

Two hundred twenty-one patients diagnosed with complete KD and 262 healthy controls were recruited from January 2021 to December 2022. KD was diagnosed according to the diagnostic criteria of the American Heart Association (AHA) (3, 29). Patients with KD attended our hospital as both outpatients with follow-ups and inpatients. The patients were of Han Chinese descent and unrelated. The healthy controls included age- and sex-matched children who visited our hospital within the same period for health examinations, presented without fever, and had no previous history of KD, infections, cardiovascular disease, autoimmune disease, or allergies. All patients with KD were treated and followed up according to the AHA guidelines. Symptoms of persistent or recurrent fever (axillary temperature ≥ 37.5 °C or rectal temperature ≥ 38.0 °C) following the initial IVIG infusion (2 g/kg) for more than 36 hours but not longer than seven days were considered IVIG resistance (3). Pediatric echocardiography was performed by experienced pediatric echocardiographers and was confirmed by two additional pediatric cardiologists; CAA was defined according to the guidelines for diagnosing and managing cardiovascular sequelae in KD (JCS/JSCS 2020) (30), and patients were followed up for at least 1 year, during which they underwent regular echocardiographic assessments. These guidelines use z-scores adjusted for body surface area, defining CAA as a coronary artery internal diameter ≥ 2.5 in either the proximal right or left main coronary artery or left anterior descending artery, persisting for more than a month after disease onset. CAA status and specific CAA were recorded based on the maximal z-scores and diameters of the coronary arteries in echocardiography reports. The z-scores were calculated using the Dallaire equations (31), and CAA was classified based on the following z-score intervals: small aneurysm, from 2.5 to 5 (non-inclusive); moderate aneurysm, from 5 to 10 (non-inclusive); and large or giant aneurysm, ≥ 10 or an internal diameter of ≥ 8 mm (30). Moreover, the time to reach the peak of CAA diameters was considered as the time at which maximum internal diameter of coronary artery was observed after illness onset, and the time of CAA persistence was defined as the time needed for CAA to return to normal appearance and size (z-score of < 2.5) in all coronary arteries as shown by echocardiography images, in addition to regaining normal cardiac function (3). Thrombotic events were diagnosed using echocardiography and confirmed using computed tomography coronary angiography. To further investigate the relationship between the severity of KD and *ITPKC* gene polymorphisms, the KD group was first divided into KD with IVIG resistance (n = 46) and KD with IVIG responsiveness (n = 175) subgroups. The KD group was also divided into KD with CAA (KD-CAA) (n = 82) and KD without CAA (KD-NCAA) (n = 139), and subgroup analyses were subsequently performed in patients with CAA according to the age, sex, CAA size, and prognosis (**Figure 1**). A total of 2 mL of peripheral venous blood sample was collected from each participant, anticoagulant EDTA-Na^2^ was added, and the samples were stored at −80°C.

This study was conducted in accordance with the principles of the Declaration of Helsinki and approved by the Ethics Review Committee of the First Affiliated Hospital of Guangxi Medical University (code number: 2021(KY-E-240)). Both patients with KD and those in the control group received detailed information regarding the purpose of the study. Written informed consent was obtained from the children’s parents or legal guardians.

### 2.2 DNA extraction and genotyping

Genomic DNA was extracted from 400 µL of blood collected from each participant using a TIANamp Blood DNA Kit (Tiangen, Beijing, China), and 1% agarose gel electrophoresis was used to determine the quality of the DNA, which was then stained with ethidium bromide. The purity of DNA was determined by taking the optical density of samples at 260 and 280 nm using a nucleic acid quantifier (Thermo NanoDrop 2000, Shimadzu). Samples were stored at −80°C until further analysis. SNaPshot (Applied Biosystems, Foster City, CA, USA) was used for genotyping the *ITPKC* rs7251246 polymorphism. The *ITPKC* gene was amplified by PCR using primers designed using Primer Premier 5.0 (Version 5.0; Premier, Canada). Primer sequences were as follows: forward, 5′-GAA ACA GCA GTG ACC AAG AC-3′ and reverse, 5′-CCT AGG CAA CAG AGT GAA AC-3′. The PCR master mix (total volume of 10 µL, including 2× Taq PCR Master Mix + template DNA to be amplified and PCR primers) was added to a 384-well plate, and amplification detection was performed in a Veriti 384-well PCR instrument (Applied Biosystems, Foster City, CA, USA). The thermal cycling conditions were as follows: denaturation at 94°C for 5 min followed by 35 cycles of denaturation at 94°C for 20 s, annealing at 60°C for 30 s, extension at 72°C for 30 s, and a final extension step at 72°C for 3 min. DNA sequencing was performed by a biotechnology company (Tianyi-Huayu Gene Sci, Wuhan, China), and an ABI 3730xl DNA Analyzer (Applied Biosystems, Foster City, CA, USA) was used to analyze the sequencing results.

### 2.3 Quantitative real-time PCR analysis

Total RNA was extracted from 500 µL of blood collected from each participant using a Blood Total RNA Kit (Simgen, Hangzhou, China). Concentration and quality of total RNA were assessed using a nucleic acid quantifier and then reverse transcribed into cDNA using the HiScript QRT SuperMix for real-time quantitative PCR (+gDNA Wiper; Vazyme, Nanjing, China). Next, real-time quantitative PCR analysis was performed using a StepOnePlus Real-Time PCR system (Applied Biosystems, Foster City, CA, USA) with SYBR® Select Master Mix (2X) under the following cycling conditions: 95°C for 30 s followed by 40 cycles of 95°C for 10 s and 60°C for 30 s. The following primers were used: *ITPKC*, forward 5′-TGC CAT CTG TCA AAT CGG-3′ and reverse 5′-CCA TCT GGG AGC AGT TAC CT-3′; GAPDH, forward 5′-GAG GCA TTG TGA ACC GCA-3′ and reverse 5′-GTG CTT CCT CCG TGT CTG T-3′. The relative amounts of all mRNAs were normalized to GAPDH mRNA expression levels using the 2^−ΔΔCT^ method, and the results are shown as fold changes relative to controls.

### 2.4 Data collection

We collected information regarding the following immune/inflammation-related indicators from children with KD: counts of white blood cell (WBC), neutrophils, and lymphocytes; platelet count; serum sodium and albumin concentrations; and C-reactive protein (CRP) concentration. Laboratory data were collected during the acute febrile period and before IVIG administration. Neutrophil-to-lymphocyte count ratio (NLR), platelet-to-lymphocyte count ratio, capillary leakage index (CLI) = CRP (mg/L)/albumin (g/L), and systemic immune-inflammation index (SII) = platelet count (×10^9^/L)× (neutrophil count [×10^9^/L]/lymphocyte count [×10^9^/L]) were calculated based on the indexes mentioned above. A routine echocardiogram was performed before IVIG treatment in the acute stage (baseline, days < 10), followed by repeated exams at 1, 3, 6, and 12 months after the onset of fever, and then on a 6-month to annual basis until the CAAs had returned to their normal size.

### 2.5 Statistical analysis

Statistical differences in allele and genotype frequencies were evaluated using Fisher’s exact test, the chi-square test, or Pearson’s chi-square test for all groups. The chi-square test was used to assess the demographic distributions and genotype frequencies in children with KD and healthy controls. The Hardy–Weinberg equilibrium was assessed using the chi-square test with 1 degree of freedom for the control group. Differences in immune/inflammation-related indicators between different genotypes among children with KD were evaluated by one-way analysis or the Kruskal–Wallis H test. Bonferroni correction was applied for multiple comparisons. The crude and adjusted odds ratios (ORs) and 95% confidence intervals (CIs) were also calculated using unconditional univariate logistic regression analysis to evaluate the associations between genotypes and KD subgroups after adjustment and stratification by age and sex. A two-tailed *P* value of < 0.05 was considered significant in statistical analyses performed using IBM SPSS Statistics for Windows, version 26 (IBM Corp., Armonk, New York, USA).

## 3 Results

### 3.1 Baseline characteristics

A total of 221 patients with KD and 262 controls were recruited in this study; 68.3% (151/221) of the KD cases and 61.1% (160/262) of the controls were male patients. The mean age of the patients and controls was 3.31 ± 2.59 years and 3.61 ± 2.46 years, respectively. No significant difference was observed between the two groups regarding age and sex (*P* = 0.097 and *P* = 0.078, respectively). Of the children with KD, 37.1% (82/221) had CAA formation, and 20.8% (46/221) had initial IVIG resistance. Details of all included studies are shown in **Supplementary Table S1**.

### 3.2 Association between *ITPKC* SNP rs7251246 and KD

We determined the genotype frequency distributions among the KD cases and controls to explore the association between the *ITPKC* rs7251246 polymorphism and KD susceptibility. As shown in **Table 1**, the genotype frequencies of the controls were within the Hardy–Weinberg equilibrium (*P* = 0.756). The genotype frequency distributions of the different *ITPKC* rs7251246 polymorphisms were 29.0% (TT), 51.6% (CT), and 19.5% (CC) in the KD group, and 30.5% (TT), 52.3% (CT), and 17.2% (CC) in the control group. No significant difference was observed between controls and children with KD or children with or without IVIG resistance regarding genotypes, alleles, and carrier frequencies of rs7251246 polymorphisms. Notably, a statistically significant difference was observed between children with and without CAA (genotype distribution, *P* = 0.046; allele frequency, *P* = 0.020; dominant model, *P* = 0.017; additive model, *P* = 0.021); Additional subgroup analyses, according to age, sex, and CAA size, did not identify differences between any subgroups; however, differences in allele frequency (*P* = 0.094), recessive model (*P* = 0.072), and additive model (*P* = 0.086) reached a borderline-significant difference between CAA with and without thrombosis. A separate sub-analysis was conducted in male children with CAA for additional prognosis analysis; a higher risk of thrombosis was observed when the CC genotype was present compared with CT/TT genotypes (recessive model, *P*=0.029). Unconditional univariate logistic regression analysis showed that the *ITPKC* SNP rs7251246 was significantly associated with CAA formation [CC/CT vs. TT: OR = 2.176, 95% CI = 1.138–4.162, *P* = 0.019]. After correcting for age and sex, the significance of rs7251246 remained [CC/CT vs. TT: adjusted OR = 2.089, 95% CI = 1.085–4.020, *P* = 0.027]. In addition, male children with the rs7251246 CT/TT genotype had a lower risk of thrombosis than male children with the CC genotype [adjusted OR = 0.251, 95% CI = 0.068–0.923, *P* = 0.037] (**Table 2**).

**Table 1:** Genotyping and allele frequency of ITPKC in KD cases and controls, as well as in KD subgroups.

rs7251246 (T/C)	Total	Genotype distribution				Allele frequency			Dominant P-value	Recessive P-value	additive P-value
		TT	CT	CC	P value	T	C	P value			
	n	n(%)	n(%)	n(%)		n(%)	n(%)				
Cohort 1 (All patients)											
Patients with KD	221	64 (29.0)	114 (51.6)	43 (19.5)	0.797	242 (54.8)	200 (45.2)	0.548	0.706	0.518	0.512
Normal control	262	80 (30.5)	137 (52.3)	45 (17.2)		297 (56.7)	227 (43.3)				
Cohort 2 (All patients with KD)											
IVIG responsiveness	175	54 (30.9)	84 (48.0)	37 (21.1)	0.095	192 (54.9)	158 (45.1)	0.775	0.358	0.114	0.477
IVIG resistance	46	11 (23.9)	30 (65.2)	5 (10.9)		52 (56.5)	40 (43.5)				
Cohort 3 (All patients with KD)											
CAA	82	16 (19.5)	46 (56.1)	20 (24.4)	**0.046**	78 (47.6)	86 (52.4)	**0.020**	**0.017**	0.155	**0.021**
NCAA	139	48 (34.5)	68 (48.9)	23 (16.5)		164 (59.0)	114 (41.0)				
Cohort 4 (KD with CAA)											
<1 year	14	3 (21.4)	8 (57.1)	3 (21.4)	0.757	14 (50.0)	14 (50.0)	0.727	1.000	0.447	0.814
1–5 years	47	9 (19.1)	29 (61.7)	9 (19.1)		47 (50.0)	47 (50.0)				
>5 years	21	4 (19.0)	10 (47.6)	7 (33.3)		18 (42.9)	24 (57.1)				
Cohort 5 (KD with CAA)											
Male	61	13 (21.3)	35 (57.4)	13 (21.3)	0.686	61 (50.0)	61 (50.0)	0.424	0.703	0.704	0.460
Female	21	3 (14.3)	12 (57.1)	6 (28.6)		18 (42.9)	24 (57.1)				
Cohort 6 (KD with CAA)											
Small-sized CAA	27	7 (25.9)	12 (44.4)	8 (29.6)	0.492	26 (48.1)	28 (51.9)	0.624	0.370	0.581	0.639
Medium-sized CAA	28	6 (21.4)	17 (60.7)	5 (17.9)		29 (41.3)	27 (58.7)				
GCAA	27	3 (11.1)	17 (63.0)	7 (25.9)		23 (42.6)	31 (57.4)				
Cohort 7 (KD with CAA)											
Thrombosis	35	5 (14.3)	18 (51.4)	12 (34.3)	0.170	28 (40.0)	42 (60.0)	0.094	0.303	0.072	0.086
No-thrombus	47	11 (23.4)	28 (59.6)	8 (17.0)		50 (53.2)	44 (46.8)				
Cohort 8 (Male patients with KD and CAA)											
Thrombosis	28	4 (14.3)	14 (50.0)	10 (35.7)	0.075	22 (39.3)	34 (60.7)	**0.044**	0.217	**0.029**	**0.035**
No-thrombus	33	9 (27.3)	20 (60.6)	4 (12.1)		38 (57.6)	28 (42.4)				

Data are expressed as frequency (percentage). The bold values highlight the statistically significant P-values (P < 0.05). ITPKC, inositol 1,4,5-trisphosphate 3-kinase C; SNP, single-nucleotide polymorphism; KD, Kawasaki disease; IVIG, intravenous immunoglobulin; CAA, coronary artery aneurysm; NCAA, no coronary artery aneurysm; GCAA, giant coronary artery aneurysm.

**Table 2:** Stratification analysis of the association between *ITPKC* rs7251246 polymorphisms and the risk of Kawasaki disease among groups.

Genotype	OR (95%CI)	*P*	Adjusted OR (95%CI)	*P^a^*
All patients (n=483)				
Controls (n=262) vs. KD (n=221)				
TT	1.000		1.000	
CT	1.040 (0.689–1.571)	0.851	1.104 (0.688–1.771)	0.681
CC	1.194 (0.702–2.033)	0.513	1.391 (0.746–2.593)	0.300
Dominant	1.078 (0.729–1.596)	0.706	1.075 (0.725–1.594)	0.718
Recessive	0.858 (0.541–1.363)	0.518	0.858 (0.539–1.367)	0.520
All patients with KD (n=221)				
IVIG responsiveness (n=175) vs. IVIG resistance (n=46)				
TT	1.000		1.000	
CT	0.581 (0.269–1.257)	0.168	0.566 (0.260–1.232)	0.152
CC	1.577 (0.506–4.914)	0.432	1.633 (0.526–5.257)	0.386
Dominant	1.382 (0.653–2.927)	0.398	1.395 (0.656–2.970)	0.387
Recessive	0.440 (0.162–1.190)	0.106	0.410 (0.149–1.127)	0.084
NCAA (n=139) vs. CAA (n=82)				
TT	1.000		1.000	
CT	2.029 (1.030–3.999)	**0.041**	1.993 (1.004–3.954)	**0.049**
CC	2.609 (1.144–5.948)	**0.023**	2.361 (1.023–5.450)	**0.044**
Dominant	2.176 (1.138–4.162)	**0.019**	2.089 (1.085–4.020)	**0.027**
Recessive	0.615 (0.313–1.206)	0.157	0.670 (0.337–1.333)	0.254
KD with CAA (n=82)				
No-thrombus (n=47) vs. Thrombosis (n=35)				
TT	1.000		1.000	
CT	1.414 (0.421–4.751)	0.575	1.523 (0.446–5.207)	0.502
CC	3.300 (0.826–13.181)	0.091	3.313 (0.803–13.665)	0.098
Dominant	1.833 (0.573–5.865)	0.307	1.927 (0.591–6.280)	0.277
Recessive	0.393 (0.140–1.104)	0.076	0.413 (0.142–1.199)	0.104
Male patients with KD and CAA (n=61)				
No-thrombus (n=33) vs. Thrombosis (n=28)				
TT	1.000		1.000	
CT	1.575 (0.404–6.146)	0.513	1.574 (0.403–6.145)	0.514
CC	5.625 (1.077–29.371)	**0.041**	5.565 (1.063–29.123)	**0.042**
Dominant	2.250 (0.609–8.311)	0.224	2.239 (0.606–8.280)	0.227
Recessive	0.248 (0.068–0.911)	**0.036**	0.251 (0.068–0.923)	**0.037**

a Logistic regression analysis adjusted for age and sex. The bold values highlight the statistically significant P-values (P < 0.05); ITPKC, inositol 1,4,5-trisphosphate 3-kinase C; KD, Kawasaki disease; IVIG, intravenous immunoglobulin; CAA, coronary artery aneurysm; NCAA, no coronary artery aneurysm.

### 3.3 Association between *ITPKC* rs7251246 SNP and immune/inflammation-related indicators and prognosis predictors

**Figure 2:**
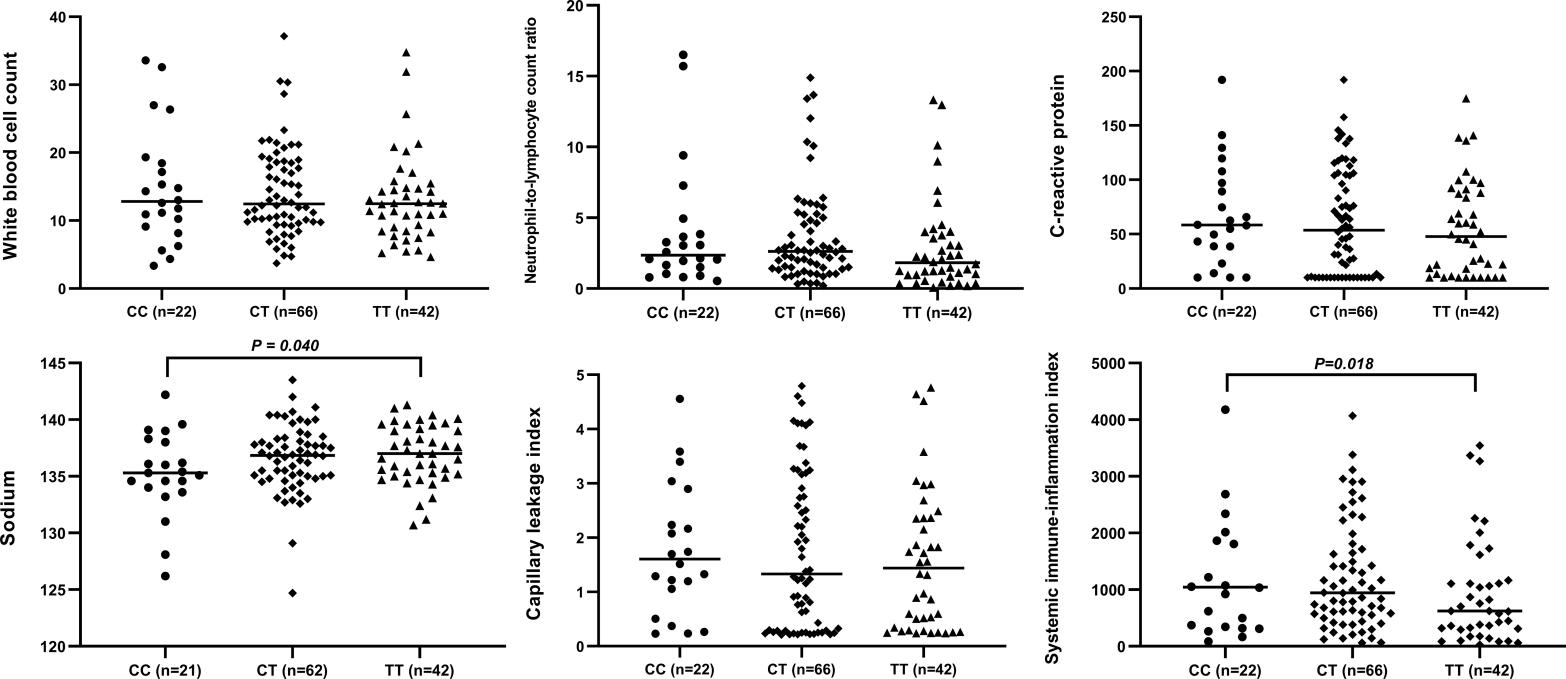
Relationship between rs7251246 genotypes and immune/inflammation-related indicators in patients with KD. KD, Kawasaki disease.

**Figure 3:**
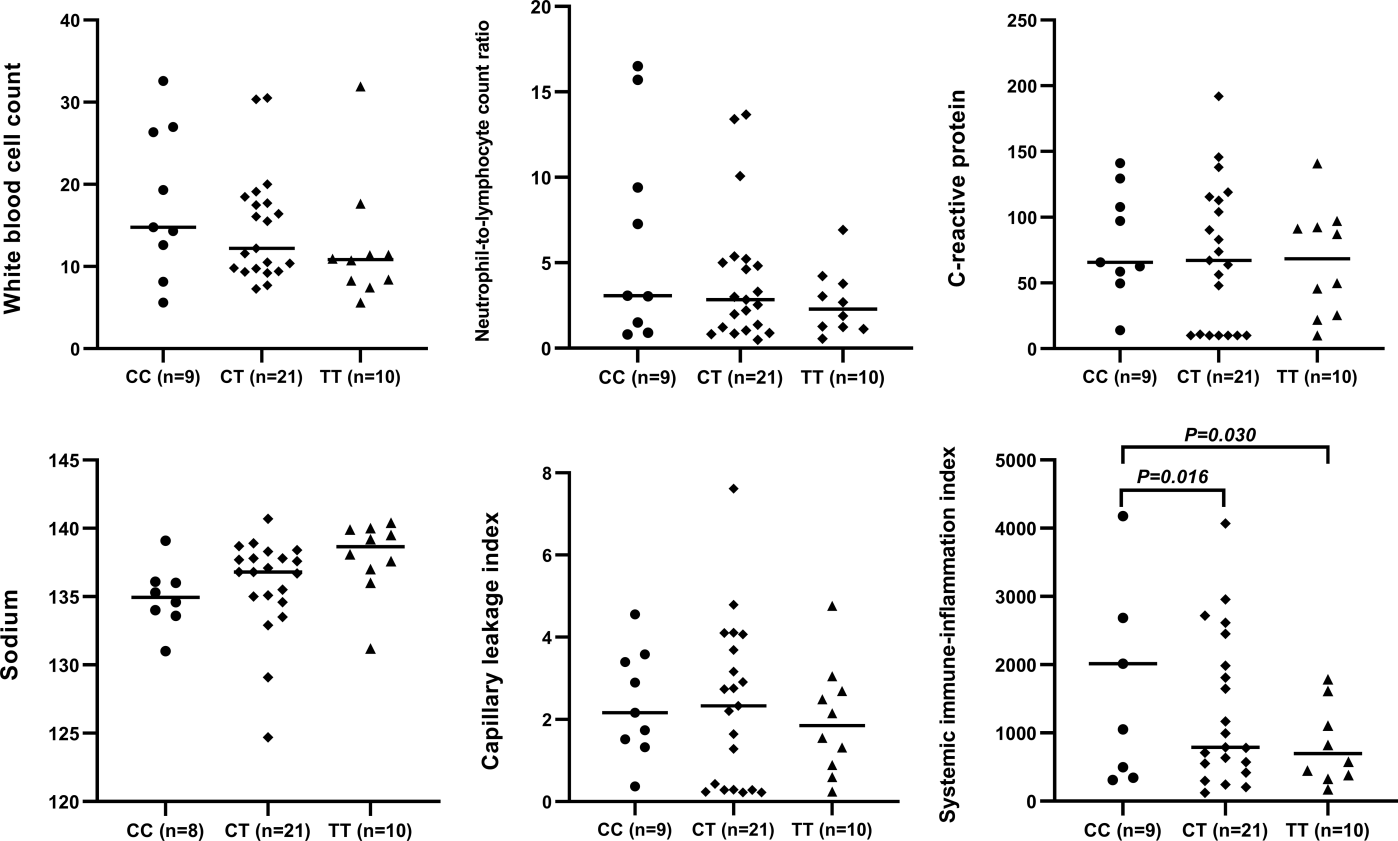
Relationship between rs7251246 genotypes and immune/inflammation-related indicators in patients with KD and CAA. KD, Kawasaki disease; CAA, coronary artery aneurysm.

**Figure 4:**
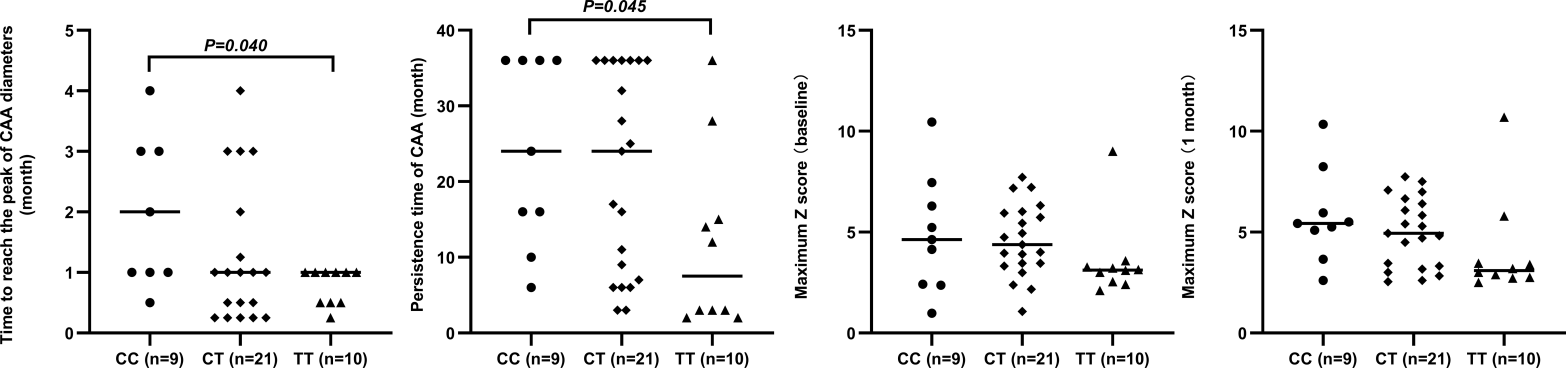
Relationship between rs7251246 genotypes and prognostic indicators in patients with KD and CAA KD. Kawasaki disease; CAA, coronary artery aneurysm.

The circulating levels of immune/inflammation-related indicators measured in the plasma of affected children during the acute phase of KD are shown in **Figure 2**. Children with the CC genotype had the highest concentrations of these indicators (WBC, NLR, CRP, CLI, and SII) and significantly lower serum sodium concentrations. Similar results were obtained for children with or without CAA, and children with the CC genotype had a significantly higher SII than those with either CT or TT genotypes (**Figure 3**). Furthermore, children with CAA with the CC genotype had longer progressive coronary diameter dilatation and the duration of aneurysm and a larger maximum z-score at baseline and one month after onset (**Figure 4**). More details on the indicators between different genotypic groups are shown in **Supplementary Table S2**.

### 3.4 Expression mRNA level of *ITPKC* in the peripheral blood

**Figure 5:**
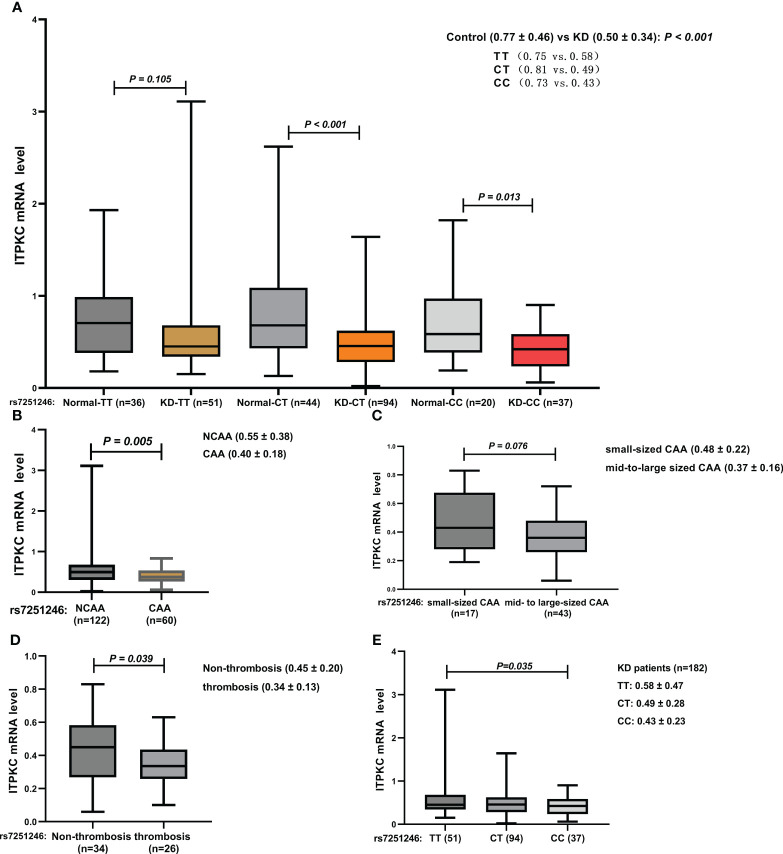
Comparison of ITPKC gene expression among the groups **(A)** KD vs. control; **(B)** KD with vs. without CAA; **(C)** small-sized CAA vs. mid- to large-sized CAA; **(D)** CAA with vs. without thrombosis; **(E)** KD with different genotypes. ITPKC, Inositol 1,4,5-trisphosphate 3-kinase C; KD, Kawasaki disease; CAA, coronary artery aneurysm.

The elevation of immune/inflammation-related indicators is associated with T cell activation; hence, the expression of *ITPKC* at the mRNA level was examined using data from whole blood serial samples obtained from children with KD (n = 182) and healthy children (n = 100). Details regarding the comparison of *ITPKC* mRNA expression among groups with different rs7251246 genotypes are shown in **Supplementary Table S3**. Significant downregulation of *ITPKC* mRNA expression was observed in children with KD (0.50 vs. 0.77, *P* < 0.001, **Figure 5**), especially in those with KD and CAA (0.40 vs. 0.55, *P* = 0.005, **Figure 5**). Furthermore, compared to those with small-sized CAA, a more robust downregulation of *ITPKC* mRNA expression was observed in children with KD and mid- to large-sized CAA (0.48 vs. 0.37, *P* = 0.076, **Figure 5**). However, the small sample size limited statistical power. Notably, the mRNA levels of *ITPKC* were significantly lower in children with CAA who developed thrombosis than in those without thrombosis (0.34 vs. 0.45, *P* = 0.039, **Figure 5**). Interestingly, among the children with KD, those with the TT genotype expressed the highest level of *ITPKC* mRNA, followed in sequence by those with CT and CC genotypes. The difference between CC and TT genotypes reached statistical significance (0.43 vs. 0.58, *P* = 0.035, **Figure 5**).

## 4 Discussion

We investigated the association between the *ITPKC* polymorphism (rs7251246) and susceptibility to KD as well as the severity of coronary artery damage in a case-control investigation of a Han Chinese population. Our study included 221 children with KD (46 with IVIG resistance and 82 with CAA) and 262 healthy controls. We found that the rs7251246 polymorphism of *ITPKC* did not influence the susceptibility to KD or response to IVIG therapy in our population, only CAA development. We found that the *ITPKC* rs7251246 T > C polymorphism is associated with coronary aneurysm complications in children with KD. Furthermore, the rs7251246 SNPs were overrepresented in patients with elevated immune/inflammation-related marker levels. Our gene expression study also found a significant downregulation of *ITPKC* mRNA expression in children with KD, especially those with CAA, compared to controls.

Despite many years of research, KD’s etiology and pathogenesis remain unknown, limiting the treatment options. Genetic susceptibility is believed to be a key factor contributing to the etiopathogenesis of KD. Several genes essential to the pathogenesis of cardiovascular diseases or the systemic inflammatory immune response have been confirmed to be involved during the onset and development of KD. The *ITPKC* gene is known to play a role in immune activation and the T-cell receptor signaling, mediating NLRP3 inflammasome activation via control of [Ca^2+^]_i_ mobilization and directing production of interleukin-1β (IL-1β) and IL-18 (18, 32, 33). Cyclosporine, an inhibitor of T cells (Ca^2+^/NFAT) pathway, can be combined with IVIG to reduce the incidence of CAA (34), pointing to calcium mobilization as a key mechanism of KD immunopathogenesis. Calcium-dependent inflammatory cytokines can be over-activated by decreasing the *ITPKC* gene expression via IP_3_-mediated pathways. Consequently, coronary vessel walls become damaged, causing formation of aneurysms (26). *ITPKC* gene polymorphisms have important functional consequences in KD, considering that treatment failure in those with the high-risk *ITPKC* genotype is suggested to be linked to higher intracellular calcium levels and increased IL-1β and IL-6 production, and higher circulating levels of both cytokines (18). Thus, surveys of SNPs associated with susceptibility to and severity of KD, the development of coronary artery complications, and IVIG resistance are ongoing (16) (see **Supplementary File 1** for the review of SNPs of the *ITPKC* gene in association with KD). A novel SNP, rs7251246, was reported to be associated with KD severity in Taiwanese patients in 2014 (28); however, it has not been investigated in a different population, and *ITPKC* expression is affected by rs7251246 in an unknown manner. In this case-control study, we investigated the association of this SNP with KD in Han Chinese children with or without CAA.

Compared to the studies by Kuo et al. that investigated the rs7251246 SNP in Taiwanese patients (28), our study used a more sensitive body surface area-adjusted coronary artery z-score to define CAA and, as a result, reported statistically significant results. We found that the *ITPKC* SNP was significantly associated with CAA formation but not with KD susceptibility or IVIG resistance, which is consistent with the results of a previous study (28) supporting the hypothesis that different genes determine susceptibility to KD and CAA formation. Remarkably, our study further identified that the *ITPKC* rs7251246 CC/CT genotype significantly increased the risk of CAA in children with KD and that the CC genotype may increase the risk of thrombus formation even further, as a borderline difference was observed between the groups.

Male sex has been recently reported to be associated with a high incidence of coronary thrombosis (35). In the present study, we found that male patients with CAA and the rs7251246 CC genotype had a significantly higher risk of thrombosis than children with the CT/TT genotype. This result may be attributed to male patients being more genetically susceptible to CAA and thrombotic events (35, 36). As low-dose aspirin alone is not sufficient to prevent CAA thrombosis (37–39), the AHA recommends dual antiplatelet therapy as prophylaxis for patients at high risk (3); hence, dual antiplatelet therapy is recommended for male children with the CC genotype of rs7251246 to prevent thrombosis because the medical management of such patients depends on the judicious use of thromboprophylaxis. To our knowledge, this study is the first to investigate the association between the *ITPKC* rs7251246 polymorphism and KD susceptibility in a pediatric Han Chinese population, as well as CAA subgroups, providing evidence for the value of risk stratification among patients with CAA, which may facilitate the identification of high-risk patients in need of targeted interventions.

Genetic studies have shown that polymorphisms of *ITPKC*, which fail to downregulate NFAT translocation in T-cells, are an important predisposing factor for KD (17, 40). Decreased *ITPKC* expression results in overly active T cell activation, leading to an excessive inflammatory response (17), which is involved in KD pathogenesis (40–42). This study showed significant downregulation of *ITPKC* mRNA expression in children with KD, especially those with KD and CAA, compared to controls. This further supports the theory that decreased *ITPKC* expression promotes KD and its serious complications (41, 42). Moreover, children with CAA and the rs7251246 CC genotype had a significantly higher SII than children with the CT/TT genotype. SII is proposed as an independent risk factor for several cardiovascular complications in patients with KD (43), suggesting that a vigorous inflammatory and host immune response may occur in these children.

Additionally, a progressive increase in CAA size and a longer duration of CAA are strongly correlated with poor long-term prognosis of coronary artery disease (30, 44–48). This study explored the mechanism by which rs7251246 affected *ITPKC* expression for the first time and presented a promising therapeutic target for KD therapy; however, the biological impact of rs7251246 in KD pathogenesis requires further study. Furthermore, the study observed markedly prolonged dilatation of coronary diameter and aneurysm persistence time in children with CAA and the rs7251246 CC genotype. These observations reiterate that these children’s severe inflammatory reactions and tissue injury warrant additional attention. Notably, *ITPKC* mRNA expression was the highest in the TT genotype and lowest in the CC genotype, and statistically significant differences were observed between the two genotypes. Based on this, we hypothesize that a reduction in the splicing efficiency of *ITPKC* mRNA due to the rs7251246 C allele might be responsible for the decrease in *ITPKC* expression. However, we cannot reach a robust conclusion if rs7251246 is a functional SNP of the *ITPKC* gene based on the available evidence, as the result does not exclude the possibility of a contribution by other polymorphisms in *ITPKC* to mRNA expression, such as rs7257602 and rs890934—both also located in intron 1 of the gene—even though no association with CALs formation was observed in the Taiwanese population (28). A more extensive array of SNPs may need to be assessed to find such an association, particularly when subjects with different ethnic backgrounds are tested.

The current study expanded on the findings of Kuo et al. and included more CAA subgroup analysis and an unconditional univariate, adjusted logistic regression analysis for the prediction of CAA and thrombosis, which ultimately could be considered the more important endpoint. Additionally, the current study also examined the mean difference of *ITPKC* mRNA expression between different genotypic groups. Several limitations, however, apply to the present study. We only enrolled patients who fulfilled the complete criteria of KD in order to maximize the homogeneity of the clinical phenotype. Furthermore, the incidence of CAA (37.1%) was substantially higher than that in previous reports (2–24.1%) because of a significant reduction in the number of children with KD recruited due to the COVID‐19 epidemic (49, 50). Most clinical specimens were obtained from outpatients with follow-ups during this period. Further, given its invasive nature, coronary angiography was only performed in patients with giant CAA or thrombosis, which did not allow for comparisons between echocardiography and coronary angiography findings. However, the results of our study are consistent with those in the study by Kuo et al. regarding the genotyping and allele frequency of rs7251246 in KD cases and controls, as well as in children with or without CAA (see **Supplementary Table S4**). Additionally, while we considered the effects of age and sex in the logistic regression analysis, our limited matching criteria may have resulted in unintended biases in the results. We did not consider other factors, such as hereditary familial factors, birth history, and environmental interactions, which may have impacted the outcome of the analysis. Another limitation is that we focused on only one allele associated with *ITPKC*, rs7251246 T > C; other polymorphisms and *ITPKC* loci were omitted. Furthermore, we only analyzed a Southern Han Chinese child population, and this study had a small sample size, which might limit the generalization of our results. Finally, we did not perform *ITPKC* rs7251246 functional studies, which are needed to confirm the relationship with the phenotype. Prospective future studies with larger sample sizes that consider additional risk factors or other ethnicities should be conducted.

## 5 Conclusions

Our results suggest that the *ITPKC* rs7251246 T > C polymorphism may be involved in the development of CAA and the severity of coronary artery damage in children with KD. This genotype could be considered a predictive biomarker of CAA in pediatric patients with KD in the Han Chinese population, especially male children. Follow-up studies are required to investigate the affected mechanisms of *ITPKC* rs7251246 T > C in children with KD.

## Data availability statement

The datasets generated and/or analyzed during the current study are available from the corresponding author upon reasonable request.

## Ethics statement

Written informed consent was obtained from the individual(s), and minor(s)’ legal guardian/next of kin, for the publication of any potentially identifiable images or data included in this article.

## Author contributions

JL drafted the manuscript, contributed to the data collection, performed the statistical analysis, provided the figures, and approved the final manuscript as submitted. PY administered primary treatment to these patients while they were admitted, contributed to the study design, and approved the final manuscript as submitted. DS contributed to the study design and performed the statistical analysis. YP and DS conceived and designed the study, contributed to the data collection, approved the final manuscript as submitted, and contributed equally to this work. All authors contributed to the article and approved the submitted version.
