# Supplementary material for: ITPKC polymorphism (rs7251246 T > C), coronary artery aneurysms, and thrombosis in patients with Kawasaki disease in a Southern Han Chinese population
Source: Front Immunol. 2023 Jun 19;14:1184162. doi: 10.3389/fimmu.2023.1184162 (PMC10315485; doi:10.3389/fimmu.2023.1184162)
Supplement: Supplementary file 1 [file Table_1.docx]

Table S1 Characteristics of the patients with Kawasaki disease and normal controls

|  | Patients with KD | Normal controls |
| --- | --- | --- |
| Characteristics | N = 221 | N = 262 |
| Male gender, No.(%) | 151 (68.3) | 160 (61.1) |
| Mean (SD) age (years) | 3.31±2.59 | 3.61±2.46 |
| Age range (years) | 0.2–13.3 | 0.2–12.1 |
| IVIG resistance, No.(%) | 46 (20.8) |  |
| CAA formation, No.(%) | 82 (37.1) |  |
| Small-sized CAA | 27 (12.2) |  |
| Medium-sized CAA | 28 (12.7) |  |
| GCAA | 27 (12.2) |  |
| Thrombosis, No.(%) | 28 (12.7) |  |

KD, Kawasaki disease; SD, standard deviation; IVIG, intravenous immunoglobulin; CAA, coronary artery aneurysm; GCAA, giant coronary artery aneurysm.
