# Supplementary material for: ITPKC polymorphism (rs7251246 T > C), coronary artery aneurysms, and thrombosis in patients with Kawasaki disease in a Southern Han Chinese population
Source: Front Immunol. 2023 Jun 19;14:1184162. doi: 10.3389/fimmu.2023.1184162 (PMC10315485; doi:10.3389/fimmu.2023.1184162)
Supplement: Supplementary file 2 [file Table_2.docx]

Table S2 Relationship between rs7251246 genotypes and indicators of the severity of inflammation and the prognosis

|  | CC genotype | | CT genotype | | TT genotype | | *P-value* |
| --- | --- | --- | --- | --- | --- | --- | --- |
| Patients with KD | | | | | | | |
| White blood cell count [×10^9^/L, ref. 5–12×10^9^/L] | 22 | 14.79±8.54 | 66 | 14.26±6.69 | 42 | 13.17±6.52 | 0.615 |
| Neutrophil-to-lymphocyte count ratio | 22 | 2.36 (1.39, 4.11) | 66 | 2.63 (1.41, 5.06) | 42 | 1.82 (0.93, 3.83) | 0.402 |
| C-reactive protein [mg/L, ref. 0–10 mg/L] | 22 | 58.40 (34.67, 99.77) | 66 | 53.60 (10.00, 104.28) | 42 | 47.81 (12.84, 88.89) | 0.598 |
| Sodium [mmol/L, ref. 137147 mmol/L] | 22 | 135.25±3.72 | 66 | 136.57±3.04 | 42 | 136.92±2.60^*^ | 0.113 |
| Capillary leakage index | 22 | 1.61 (0.92, 2.93) | 66 | 1.33 (0.29, 3.17) | 42 | 1.44 (0.33, 2.40) | 0.812 |
| Systemic immune-inflammation index | 22 | 1045.41  (336.88, 2094.61) | 66 | 944.91  (537.33, 1738.64) | 42 | 624.83  (305.70, 1277.31)^*^ | 0.170 |
| Patients with KD and CAA | | | | | | | |
| White blood cell count [×10^9^/L, ref. 5–12×10^9^/L] | 9 | 17.86±9.13 | 21 | 14.71±6.60 | 10 | 12.38±7.59 | 0.289 |
| Neutrophil-to-lymphocyte count ratio | 9 | 3.08 (1.21, 12.56) | 21 | 2.84 (1.13, 5.11) | 10 | 2.29 (1.21, 3.89) | 0.451 |
| C-reactive protein [mg/L, ref. 0–10 mg/L] | 9 | 65.82 (54.23, 118.59) | 21 | 67.22 (10.00, 114.23) | 10 | 68.46 (24.70, 93.64) | 0.802 |
| Sodium [mmol/L, ref. 137–147 mmol/L] | 9 | 134.96±2.34 | 21 | 135.89±3.60 | 10 | 137.89±2.76 | 0.136 |
| Capillary leakage index | 9 | 2.17 (1.42, 3.49) | 21 | 2.33 (0.29, 3.88) | 10 | 1.85 (0.82, 2.78) | 0.821 |
| Systemic immune-inflammation index | 9 | 2013.10  (421.00, 6720.50) | 21 | 791.12  (485.30, 2219.16)^*^ | 10 | 699.16  (367.78, 1656.33)^*^ | 0.347 |
| Time to reach the peak of CAA diameters [month] | 9 | 2.00 (1.00, 3.50) | 21 | 1.00 (0.38, 3.00) | 10 | 1.00 (0.50, 1.00)^*^ | 0.091 |
| Time of CAA persistence [month] | 9 | 24.00 (13.00, 36.00) | 21 | 24.00 (6.50, 36.00) | 10 | 7.50 (2.75, 18.25)^*^ | 0.093 |
| Maximum Z-score (Baseline) | 9 | 4.63 (2.40, 6.88) | 21 | 4.38 (3.39, 5.98) | 10 | 3.12 (2.52, 3.35) | 0.331 |
| Maximum Z-score (1 month) | 9 | 5.43 (4.38, 7.10) | 21 | 4.94 (3.25, 6.53) | 10 | 3.10 (2.75, 4.05) | 0.195 |

Data are expressed as means with standard deviations or medians (IQR).

*Statistically significant versus CC genotype. KD, Kawasaki disease; CAA, coronary artery aneurysm.
