# Supplementary material for: ITPKC polymorphism (rs7251246 T > C), coronary artery aneurysms, and thrombosis in patients with Kawasaki disease in a Southern Han Chinese population
Source: Front Immunol. 2023 Jun 19;14:1184162. doi: 10.3389/fimmu.2023.1184162 (PMC10315485; doi:10.3389/fimmu.2023.1184162)
Supplement: Supplementary file 3 [file Table_3.docx]

Table S3 Comparison of ITPKC mRNA expression among groups with different rs7251246 genotypes

|  | CC genotype | | CT genotype | | TT genotype | | *P-value* | CC vs. CT *P-value* | CC vs. TT *P-value* | CT vs. TT *P-value* |
| --- | --- | --- | --- | --- | --- | --- | --- | --- | --- | --- |
| Normal controls | 20 | 0.59 (0.39, 0.97) | 44 | 0.68 (0.43, 1.09) | 36 | 0.71 (0.38, 0.99) | 0.759 | 0.528 | 0.898 | 0.550 |
| Patients with KD | 37 | 0.42 (0.24, 0.59) | 94 | 0.45 (0.28, 0.62) | 51 | 0.45 (0.34, 0.68) | 0.090 | 0.360 | 0.035 | 0.108 |
| Patients with KD and IVIG resistance | 5 | 0.18 (0.08, 0.25) | 28 | 0.44 (0.24, 0.60) | 9 | 0.45 (0.30, 1.23) | 0.014 | 0.008 | 0.005 | 0.458 |
| Patients with KD and CAA | 16 | 0.33 (0.25, 0.59) | 32 | 0.36 (0.26, 0.50) | 12 | 0.42 (0.29, 0.57) | 0.715 | 0.951 | 0.512 | 0.427 |
| Patients with CAA and thrombosis | 8 | 0.27 (0.20, 0.41) | 14 | 0.34 (0.27, 0.48) | 4 | 0.40 (0.28, 0.45) | 0.686 | 0.472 | 0.452 | 0.801 |

Data are expressed as medians (IQR). ITPKC, inositol 1,4,5-trisphosphate 3-kinase C; KD, Kawasaki disease; IVIG, intravenous immunoglobulin;

CAA, coronary artery aneurysm.
