# Supplementary material for: ITPKC polymorphism (rs7251246 T > C), coronary artery aneurysms, and thrombosis in patients with Kawasaki disease in a Southern Han Chinese population
Source: Front Immunol. 2023 Jun 19;14:1184162. doi: 10.3389/fimmu.2023.1184162 (PMC10315485; doi:10.3389/fimmu.2023.1184162)
Supplement: Supplementary file 4 [file Table_4.docx]

Table S4 Genotype and allele frequencies of SNP rs7251246 in present study and study by Kuo et al.

|  | Patients with KD and controls | | | | Patients with KD with and without CAA | | | |
| --- | --- | --- | --- | --- | --- | --- | --- | --- |
|  | Kuo et al | | Present study | | Kuo et al | | Present study | |
|  | Case  (n=381) (%) | Controls  (n=569) (%) | Case  (n=221) (%) | Controls  (n=262) (%) | CAA  (n=64) (%) | NCAA (n=310) (%) | CAA (n=82) (%) | NCAA (n=139) (%) |
| TT genotype | 27.5 | 28.0 | 29.0 | 30.5 | 10.9 | 30.4 | 19.5 | 34.5 |
| CT genotype | 52.7 | 49.6 | 51.6 | 52.3 | 67.2 | 49.8 | 56.1 | 48.9 |
| CC genotype | 19.8 | 22.4 | 19.5 | 17.2 | 21.9 | 19.8 | 24.4 | 16.5 |
| *P-*value | NA | | 0.797 | | NA | | 0.046 | |
| T allele | 53.9 | 52.8 | 54.8 | 56.7 | 44.5 | 55.3 | 47.6 | 59.0 |
| C allele | 46.1 | 47.2 | 45.2 | 43.3 | 55.5 | 44.7 | 52.4 | 41.0 |
| *P-*value | 0.6411 | | 0.548 | | 0.0267 | | 0.020 | |
| Dominant *P*-value | 0.8892 | | 0.706 | | 0.0015 | | 0.017 | |
| Recessive *P*-value | 0.3396 | | 0.518 | | 0.7072 | | 0.155 | |

NA, not applicable; KD, Kawasaki disease; CAA, coronary artery aneurysm; NCAA, no coronary artery aneurysm.
