# Supplementary material for: ITPKC polymorphism (rs7251246 T > C), coronary artery aneurysms, and thrombosis in patients with Kawasaki disease in a Southern Han Chinese population
Source: Front Immunol. 2023 Jun 19;14:1184162. doi: 10.3389/fimmu.2023.1184162 (PMC10315485; doi:10.3389/fimmu.2023.1184162)
Supplement: Supplementary file 5 [file DataSheet_1.docx]

Supplementary File 1 Review of published literature on SNPs of the *ITPKC* gene in association with susceptibility to KD risk

| Study (year) and country | No. of subjects  (cases/controls) | Allele (%) in KD | Risk of KD | Risk of IVIG resistance | Risk of CALs | Other risks |
| --- | --- | --- | --- | --- | --- | --- |
| **rs28493229** | | | | | | |
| Onouchi et al. (2008), Japan ^[1]^ | 637/1034 | C = 22%;  G = 78% | Present with C allele | NA | Present with C allele | Family history |
| Chi et al. (2010), Taiwan ^[2]^ | 385/1158 | C = 8%;  G = 92% | No association | NA | No association | NA |
| Lin et al. (2011), Taiwan ^[3]^ | 280/492 | C = 8%;  G = 92% | Present with C allele | NA | No association | BCG site erythema |
| Kuo et al. (2011), Taiwan ^[4]^ | 334/1131 | C = 8.1%;  G = 91.9% | No association | No association | No association | Meta-analysis: all Taiwanese study (1998/5562):  odds ratio, 1.36; 95% confidence interval, 1.12–1.66 |
| Khor et al. (2011),  European and Asian ^[5]^ | 2173/9383 | GWAS:  C 20%, G 80%;  Taiwan:  C 8%, G 92%;  Korea:  C13%, G 87% | Present with C allele | NA | Present with C allele | Family history |
| Peng et al. (2012), China ^[6]^ | 223/318 | C = 6%;  G = 94% | No association | No association | No association | NA |
| Onouchi et al. (2013), Japanese ^[7]^ | 204/947 | C = 23.8%;  G = 76.2% | Present in C allele | No association | Present in the combined analysis only | NA |
| Yan et al. (2013), China ^[8]^ | 358/815 | C = 6.4%;  G = 93.6% | No association | NA | No association | Significant role of rs1801274 (FCGR2A), rs2857151 (HLA), and rs2254546 (BLK) in KD |
| Natividad (2013), Philippines ^[9]^ | 17/26 | C = 20.0%;  G = 40.7% | No association | NA | No association | Higher % of risk allele “C” among controls than in case postulated as protective |
| Kuo et al. (2013), Taiwanese ^[10]^ | 340/control | CC = 0.68%  CG + GG = 99.32% | No association | No association | No association | Overrepresentation of CC in IVIG nonresponders |
| Wang et al. (2014), China ^[11]^ | 428/493 | C = 7%;  G = 93% | No association | NA | No association | Combined rs2720378 (GC/CC) (CASP3), rs2069762 (CA/CC) (IL-2), and rs1561876 AA (STIM1) have higher KD risk |
| Kim et al. (2018), Korea ^[12]^ | 299/210 | C = 12%;  G = 88% | Present with CG or CC genotype | NA | Present with CG or CC genotype | ITPKC rs2561531 CC and SLC11A1 rs 17221959 CT—may exert a protective effect |
| Bhattarai et al. (2022), India ^[13]^ | 50/50 | C = 11%;  G = 89% | No association | NA | No association | NA |
| **rs2290692** | | | | | | |
| Peng et al. (2012), China ^[6]^ | 223/318 | C = 52.47%;  G = 47.53% | Present with the ancestral C allele | Overrepresentation of CC in IVIG non-responders | Present with the ancestral C allele | NA |
| Kuo et al. (2014), Taiwanese ^[14]^ | 381/569 | C = 54.4%;  G = 45.6% | No association | NA | Present with the ancestral C allele | rs7251246/rs890934/rs10420685/rs2607420/rs2290692 (C/G/G/T/G) haplotype had a significant association with CAL formation compared to the T/T/A/T/C haplotype |
| Kim et al. (2018), Korea ^[12]^ | 299/210 | C = 46.3%;  G = 53.7% | No association | NA | No association | rs2290692 SNPs were overrepresented in patients with high inflammatory markers |
| Bhattarai et al. (2022), India ^[13]^ | 50/50 | C = 52%;  G = 48% | Present with the G allele | NA | No association | NA |
| **rs10420685** | | | | | | |
| Wang et al. (2014), China ^[11]^ | 428/493 | A = 82.20%;  G = 17.80% | No association | NA | No association | Combined rs2720378 (GC/CC) (CASP3), rs2069762 (CA/CC) (IL-2), and rs1561876 AA (STIM1) have higher KD risk |
| Kuo et al. (2014), Taiwanese ^[14]^ | 381/569 | A = 79.20%;  G = 20.80% | No association | NA | No association | rs7251246/rs890934/rs10420685/rs2607420/rs2290692 (C/G/G/T/G) haplotype had a significant association with CAL formation compared to the T/T/A/T/C haplotype |
| Kim et al. (2018), Korea ^[12]^ | 299/210 | A = 71.57%;  G = 28.43% | No association | NA | NA | Combined rs10420685 (GG) and rs17235409 (AA) were strongly associated with BCG injection site erythema in children more than 2 years |
| **rs7251246** | | | | | | |
| Kuo et al. (2014), Taiwanese ^[14]^ | 381/569 | T = 53.9%;  C = 46.1% | No association | NA | Overrepresentation of CC or CT genotype | rs7251246/rs890934/rs10420685/rs2607420/rs2290692 (C/G/G/T/G) haplotype had a significant association with CAL formation compared to the T/T/A/T/C haplotype |
| **rs890934** | | | | | | |
| Kuo et al. (2014), Taiwanese ^[14]^ | 381/569 | G = 55.1%;  T = 44.9% | No association | NA | Overrepresentation of ancestral G allele | rs7251246/rs890934/rs10420685/rs2607420/rs2290692 (C/G/G/T/G) haplotype had a significant association with CAL formation compared to the T/T/A/T/C haplotype |
| **rs2607420** | | | | | | |
| Kuo et al. (2014), Taiwanese ^[14]^ | 381/569 | T = 75.1%;  C = 24.9% | Overrepresentation of CC genotype | NA | No association | rs7251246/rs890934/rs10420685/rs2607420/rs2290692 (C/G/G/T/G) haplotype had a significant association with CAL formation compared to the T/T/A/T/C haplotype |
| **rs11673492** | | | | | | |
| Kuo et al. (2014), Taiwanese ^[14]^ | 381/569 | C = 72.3%;  T = 27.7% | Overrepresentation of TT genotype | NA | No association | NA |
| **rs7257602** | | | | | | |
| Kuo et al. (2014), Taiwanese ^[14]^ | 381/569 | G = 50.10%;  A = 49.90% | No association | NA | No association | NA |
| **rs2561531** | | | | | | |
| Kim et al. (2018), Korea ^[12]^ | 299/210 | C = 78.09%;  T = 21.91% | No association | NA | NA | Combined rs2561531 (CC), rs17221959 (CT) and rs77624405 (GA) have lower KD risk in children under 2 years |

NA, not applicable; SNP, single-nucleotide polymorphism; ITPKC, inositol 1,4,5-triphosphate 3-kinase C; KD, Kawasaki disease; IVIG, Intravenous immunoglobulin; CAL coronary artery lesion; BCG, Bacille Calmette-Guerin; GWAS genome-wide association studies.

References

1. Onouchi Y, Gunji T, Burns JC, Shimizu C, Newburger JW, Yashiro M, et al. ITPKC functional polymorphism associated with Kawasaki disease susceptibility and formation of coronary artery aneurysms. Nat Genet. 2008;40(1):35-42. [https://doi.org/10.1038/ng.2007.59](https://doi.org/10.1136/archdischild-2014-307536)

2. Chi H, Huang FY, Chen MR, Chiu NC, Lee HC, Lin SP, et al. ITPKC gene SNP rs28493229 and Kawasaki disease in Taiwanese children. Hum mol genet. 2010;19(6):1147-51. [https://doi.org/10.1093/hmg/ddp586](https://doi.org/10.1136/archdischild-2014-307536)

3. Lin MT, Wang JK, Yeh JI, Sun LC, Chen PL, Wu JF, et al. Clinical Implication of the C Allele of the ITPKC Gene SNP rs28493229 in Kawasaki Disease: Association With Disease Susceptibility and BCG Scar Reactivation. Pediatr infect dis j. 2011;30(2):148-52. [https://doi.org/10.1097/INF.0b013e3181f43a4e](https://doi.org/10.1136/archdischild-2014-307536)

4. Kuo HC, Yang KD, Juo SH, Liang CD, Chen WC, Wang YS, et al. ITPKC single nucleotide polymorphism associated with the Kawasaki disease in a Taiwanese population. PLoS One. 2011;6(4):e17370. [https://doi.org/10.1371/journal.pone.0017370](https://doi.org/10.1136/archdischild-2014-307536)

5. Khor CC, Davila S, Breunis WB, Lee YC, Shimizu C, Wright VJ, et al. Genome-wide association study identifies FCGR2A as a susceptibility locus for Kawasaki disease. Nat Genet. 2011;43(12):1241-6. [https://doi.org/10.1038/ng.981](https://doi.org/10.1136/archdischild-2014-307536)

6. Peng Q, Chen C, Zhang Y, He H, Wu Q, Liao J, et al. Single-nucleotide polymorphism rs2290692 in the 3'UTR of ITPKC associated with susceptibility to Kawasaki disease in a Han Chinese population. Pediatr cardiol. 2012;33(7):1046-53. [https://doi.org/10.1007/s00246-012-0223-x](https://doi.org/10.1136/archdischild-2014-307536)

7. Onouchi Y, Suzuki Y, Suzuki H, Terai M, Yasukawa K, Hamada H, et al. ITPKC and CASP3 polymorphisms and risks for IVIG unresponsiveness and coronary artery lesion formation in Kawasaki disease. Pharmacogenomics J. 2013;13(1):52-9. [https://doi.org/10.1038/tpj.2011.45](https://doi.org/10.1136/archdischild-2014-307536)

8. Yan Y, Ma Y, Liu Y, Hu H, Shen Y, Zhang S, et al. Combined analysis of genome-wide-linked susceptibility loci to Kawasaki disease in Han Chinese. Hum Genet. 2013;132(6):669-80. [https://doi.org/10.1007/s00439-013-1279-2](https://doi.org/10.1136/archdischild-2014-307536)

9. Natividad MF, Torres-Villanueva CA, Saloma CP. Superantigen involvement and susceptibility factors in Kawasaki disease: profiles of TCR Vβ2+ T cells and HLA-DRB1, TNF-α and ITPKC genes among Filipino patients. Int J Mol Epidemiol Genet. 2013;4(1):70-6. PMID:23565324

10. Kuo HC, Hsu YW, Wu CM, Chen SH, Hung KS, Chang WP, et al. A replication study for association of ITPKC and CASP3 two-locus analysis in IVIG unresponsiveness and coronary artery lesion in Kawasaki disease. PLoS One. 2013;8(7):e69685. [https://doi.org/10.1371/journal.pone.0069685](https://doi.org/10.1136/archdischild-2014-307536)

11. Wang W, Lou J, Zhong R, Qi YQ, Shen N, Lu XZ, et al. The roles of Ca2+/NFAT signaling genes in Kawasaki disease: single- and multiple-risk genetic variants. Sci Rep. 2014;4(null):5208. [https://doi.org/10.1038/srep05208](https://doi.org/10.1136/archdischild-2014-307536)

12. Kim KY, Bae YS, Ji W, Shin D, Kim HS, Kim DS. ITPKC and SLC11A1 Gene Polymorphisms and Gene-Gene Interactions in Korean Patients with Kawasaki Disease. Yonsei med j. 2018;59(1):119-27. [https://doi.org/10.3349/ymj.2018.59.1.119](https://doi.org/10.1136/archdischild-2014-307536)

13. Bhattarai D, Kumrah R, Kaur A, Kaur A, Srivastava P, Rawat A, et al. Association of ITPKC gene polymorphisms rs28493229 and rs2290692 in North Indian children with Kawasaki disease. Pediatr res. 2022;92(4):1090-8. [https://doi.org/10.1038/s41390-021-01830-x](https://doi.org/10.1136/archdischild-2014-307536)

14. Kuo HC, Hsu YW, Lo MH, Huang YH, Chien SC, Chang WC. Single-nucleotide polymorphism rs7251246 in ITPKC is associated with susceptibility and coronary artery lesions in Kawasaki disease. PLoS One. 2014;9(3):March 12, 2014. [https://doi.org/10.1371/journal.pone.0091118](https://doi.org/10.1136/archdischild-2014-307536)
